# A Journey Through the Land of DAAP: Including Visits to Weighted Dictionaries, Smoothing, Covariations, and the Effects of Word Order, with Connections to Psychology, Psycholinguistics, Mathematics and Statistics, and Ending at Time-Based DAAP (TDAAP)

**DOI:** 10.1007/s10936-025-10145-5

**Published:** 2025-05-08

**Authors:** Bernard Maskit, Wilma Bucci

**Affiliations:** 1 Stony Brook University, Stony Brook, NY USA; 2 Department of Psychology at Adelphi University, Adelphi University, Garden City, NY USA

**Keywords:** DAAP, Weighted dictionaries, Power of function words, Smoothing and foldovers, Word order, TimeDAAP

## Abstract

This is a condensed version of a series of talks given by Bernard Maskit at the Referential Process Workshop Conference, at the New York Psychoanalytic Society and Institute (July, 2023). Dr. Maskit discusses the Discourse Attributes Analysis Program (DAAP) which he created to evaluate psychotherapy and other texts utilizing measures of the Referential Process, a theory developed in the context of multiple code theory (Bucci in Psychoanalysis and cognitive science: a multiple code theory, Guilford Press, New York, 1997, 2023). Dr. Maskit’s emphasis is for his audience to understand how his system can be used technically and conceptually, and to provide updates on how measures can be aligned with time to produce new insights. In the first days of the Workshop, Dr. Maskit reviewed the development of the DAAP and its measures; sections of these talks are summarized here with references to relevant publications. He then went on to focus on development of the new TimeDAAP and its crucial role in the Referential Process project. We note with sadness that Dr. Maskit died before he could review and edit his presentation for publication. Wilma Bucci participated in the work presented here and prepared Dr. Maskit’s talks for publication. We thank Sean Murphy, Michael Peral and Perry Suskind for their invaluable contributions to this paper.

For many people, DAAP is a black box; you feed it some text and it spits out a large quantity of data in different forms. The goal here is to help people understand how DAAP works. There are three different levels of explanation for a complicated program like DAAP. The first level, which I’ll aim at here is to explicate what DAAP does in non-technical terms. The second level, which is my natural level, is to use mathematical formulae as explanations. The third level consists of explication of the computer code; it is often quite difficult to translate the mathematics into code and conversely, to translate the code into mathematics. These explanations are not meant as user’s guides, telling you which buttons to push and how to interpret the output, but as providing a general background for understanding the goals of the project and its methods. Each version of DAAP has its own user’s guide.

## What does DAAP do?

Most text analysis systems read a text, compare each word with a set of word lists, called dictionaries, and primarily report the proportion of words in the text that match words in each of the dictionaries. DAAP does all that and does a lot more. To understand what sets DAAP apart, one has to understand the role of weighted dictionaries. These are lists of words where each word has a weight associated with it; the weight is a number between − 1 and + 1 (we also sometimes transform these weights so that they lie between 0 and + 1). DAAP uses these weights to produce data on several levels. DAAP also produces graphs showing the changing landscape of the text in terms of the Referential Process (RP) variables and others. To generate the graphs, DAAP begins by reproducing the text without standard punctuation or capitalization and including repeated syllables and indications of hesitations and other phonetic markers, then inserting numeric markers to help associate the graphs with the text, as you will see in the text examples included in these talks. DAAP then produces measures that describe characteristics of the segment or of a whole text in both numeric and graphic form. As we will discuss further, these measures depend not only on the words of the text, but also on the order in which the words are presented.

## Wilma’s Discovery

Before describing the operation of DAAP, I want to say a little about its origins. The DAAP story begins with Wilma’s discovery of a connection between a certain style of language, which she named referential activity (RA) and certain kinds of hand movements. She found that people whose language was more vivid and imaginative were also more likely to accompany their speech with hand gestures and body movements that were associated with speech rhythms and intonation patterns (Bucci, 1984; Bucci & Freedman, 1978). This language style was described in the famous style book of Strunk and White (1972); one of their rules of good writing is to “prefer the specific to the general, the definite to the vague, the concrete to the abstract.” Based on this rule, she developed the four RA scales, Concreteness, Specificity, Clarity, and Imagery; the scoring procedure was formalized in a manual (Bucci et al., 1992; Bucci & McKay, 2014). Judges rated passages of text on these four dimensions on a scale of 0 to 10; an overall RA score was generated as the mean of these four scaled scores. It is important to note that the judges rated whole passages of text, not individual words. We will return to this important point. Many students were trained in this scoring procedure, and many research studies, including many dissertations, were carried out using these scales.

The scoring was reliable but time consuming; Wilma’s wish to have automatized scoring, particularly for large texts such as therapy sessions, as well as large sample studies, left her with the problem of having to develop a computerized system of scoring a dimension such as RA, which is based on language style rather than content.

## The First Computerized Measure of RA; the CRA of Mergenthaler and Bucci

Erhard Mergenthaler, working with Wilma (1999), first addressed this problem, using large sets of many different types of texts including early memories, dreams, and monologues, as well as different types of written texts, and transcripts of parts of therapy sessions that had been scored following the RA scoring manual. They found that certain words were used more frequently in high RA speech than otherwise, and certain other words were used more frequently in low RA speech than otherwise, thus producing two dictionaries, or lists of words, that comprised computerized referential activity (CRA).

These CRA lists were used to solve the problem of an automatic method for obtaining an RA score for a segment, computed as the number of high CRA words minus the number of low CRA words, as run on Mergenthaler’s TAS (Text Analysis System). The TAS method provided useful information about overall language style in a text independent of content. However, the method used standard scores, which interfered with comparing the level of scores in different texts with one another, and reported scores as word blocks of about 150 words each. This method made it difficult to provide graphs that could be compared with the words of a text to see precisely the points where changes in style occurred.

## A Personal Note

Wilma asked me if I could find a way to solve the problem of graphically representing the ever-changing degree to which the speaker is using this kind of language, rather than relying on word blocks. I had reached a point in my career as a pure mathematics researcher where I was no longer finding problems that I could solve that were just hard enough to be worth solving and publishing in first rate journals. So I was looking for new problems and new directions.

I spent some months feeling that Wilma’s problem was impossible, and then hit on a way to solve it. The basic idea is to use what we now call DAAP smoothing, which is quite mathematical; I’ll try to describe it later. First, in order to use it, we need dictionaries that give us more of a spread of information than we got from the two levels of the CRA. That is, we looked for words that were indicative of *different levels* of RA speech. This opened the need for the weighted dictionaries that became central to our project.

## Why Should you Make a Weighted Dictionary and How do you do it?

The gist of Wilma’s initial discovery is that RA is a psychological process, and one whose manifestations is the language style that we associate with narrative or more generally, symbolizing. The goal of the WRAD (Weighted Referential Activity Dictionary) is to capture the different levels of this RA style and usage. Generalizing Mergenthaler and Bucci (1999), we looked at RA scores of segments, and then tried to model these scores using a weighted dictionary; a set of words, each with a weight that is indicative of the RA scale score of segments in which this word is primarily used. Crucially, this rating process had been developed through judgments by trained raters based on their reactions to whole passages—not cognitive judgments, but reactions based on feelings that the passages evoked.

### An Example of High WRAD Text

We were in a different town. and, i can remember we di- we didn’t have a phone at the time and we’d had a a next door neighbor had a phone, and, they came round and said ”will you come, your er, father’s on the phone” and, c__ (husband) had gone round and he came back and said er ”your mother’s in hospital, they’ve had to take her in” and it was my c__ (husband) ’s mother who had gone round that particular day and knocked on the door, and my mother had actually come down stairs and had collapsed at the door. and it was she who had rung the doctor and they’d rushed her into hospital. and she said you know “there was absolutely no food in that house at all” he didn’t know how to cope with her, because she’d always, he’d never done anything. and i can remember saying at the time, “i’ll never forgive him, (x mm) if she dies” and she did. you know, i felt that, then, that that is one time when i really did (P yeah) feel strongly. because i felt he’d neglected her.

### An Example of Low WRAD Text

i don’t think it matters now does it? (laughs) (T mm) yes, i mean you’ve got that sort of er, you’d be very good on a a a team game, you know, where you, had to guess the one that was telling the truth, because you don’t give anything away (laughs) on your face at all. you know, an inscrutable. i’m not saying (P mm hm) that you should do, i mean you are there, in a way, you’re here as a interpreter aren’t you? instead of interpreting languages, you’re interpreting, what, i say (P mm-hm) and looking for clues, an investigator or whatever you like.

A related but somewhat different technique was used for constructing the Italian version of the WRAD (Mariani et al., 2013). This new technique was used for the development of all subsequent weighted dictionaries. There is also a Spanish WRAD, constructed by Andres Roussos. In addition, a group in Israel has constructed a Hebrew WRAD by translating from the English version.

## Constructing the WRRL

At this point, we’ve made several weighted dictionaries: the Weighted Referential Activity Dictionary (WRAD); the Italian WRAD (IWRAD); the Weighted Reflecting/Reorganizing List (WRRL); the Italian WRRL (IWRRL); and the Weighted Arousal List (WRSL). These were all constructed using similar techniques. Details concerning the construction of WRAD (Bucci and Maskit, 2006) and IWRAD (Mariani et al., 2013) have been published. Some details concerning the construction of the WRRL have also been published (Zhou et al., 2021). Without going into too much detail, I’ll describe the construction of the WRRL. Karen Tocatly’s paper (in this issue, 2025) discusses the WRSL.

We gathered segments from various sources, with segments generally being between 100 and 300 words long. To start the process, a set of about 10 segments was distributed to individual members of the team. Starting with a general idea of what the Reflecting/Reorganizing (RR) dimension was about, we each individually scored the segment for the RR dimension, on a scale of 0 to 10, without a definition of this dimension having as yet been formally stated.

The scores were discussed, and the process repeated several times so that a definition of the dimension and reliability of scoring was gradually reached. The two most reliable raters scored the remaining segments; their scores were averaged to obtain a RR score for each segment.

The construction of the WRRL (as well as the WRSL, to be described by Tocatly, 2025) differed from the construction of the WRAD, in that the work essentially had to start from scratch. We did not begin with a basic scoring manual. We did not have a relevant analysis of language style as Strunk and White (1972) had provided. We did not even have a systematic theoretical definition of the dimension to begin with. We essentially had to build the definition of the dimension as part of developing the measure and the scoring instructions.

Eventually, through the iterative, interactive method that I have described, we developed a weighted dictionary that captured the qualities of language that are produced when people are engaged in self-examination and emotional change. This method contrasts with the previously used unweighted *Reflection* dictionary that indicates a more intellectual process, as Zhou et al. (2021) describe in detail. I note that this bootstrapping procedure was also used in development of the Weighted Arousal List (WRSL). For the WRRL and WRSL, as for the WRAD, segments were scored, not individual words, and the score of a segment depends not only on the words in the segment but the order in which the words are presented (more on this later).

The segments scored for WRRL were separated into two sets: a Dictionary set, consisting of 266 segments and a Test Set consisting of 30 segments. The 30 items in the Test set were chosen randomly within the constraint that each of the 30 segments was required to have a different speaker or author.

### A High RR Segment

One meaning of it is the analysis. You multiply everything; everything is multiplied but it’s you’re with the man this image the female keeps trying to intrude and you want her to stay the hell out because it’s too frightening. there seem to be two kinds of dangers you’ve been through one is she takes something from you; she takes the shit she takes the penis and the second is you do something very violent and filthy and dirty and destructive.

### A Low RR Example

he said, ”you know your niece.” i said of course i know my niece and he said uhm, uhm, now my brother came last night and brought me flowers and he didn’t tell me any of this and i – i said, you know i don’t know what the hell this was and he says well you know ”she won’t even go with your brother anywhere anymore.” so i was like uh why, and he says well ”probably because of the epilepsy, you know.” what am i doing? and then it was over. but i wouldn’t really take my word because i just don’t.

## Into the World of Computer Algorithms: Going from Scores for Segments to Scores for Words

To develop the weighted dictionaries, a list of all the words (types) appearing in at least two of the dictionary segments was compiled. For each of these words (types), a list of the dictionary scores of segments in which the word appears was compiled (tokens). This included repetitions, so that if a word appeared twice in a segment scored x by the raters, x appeared twice in the list of scores for this word.

A word was included in the dictionary if the distribution of scores was sufficiently modal (like a normal curve) and not too flat. The median of these scores was recorded as its dictionary weight along with the word. But, how do you know if the distribution of scores is sufficiently modal?

## Supervised Machine Learning

A measure of flatness is given by the absolute difference between the First Quartile (Q_1_) and Third Quartile Q_3_, S =|Q_1_ − Q_3_|. By definition, half the scores fall in the range between Q_1_ and Q_3_. Since each score lies between 0 and 10, the possible range of values for S is between 0 and 10. For each of the 40 numbers at intervals of 1/4 between 0 and 10, call the number L, a dictionary was constructed where a word was entered in the dictionary with weight equal to the median M, consisting of all words whose spread S <  = L. This L dictionary was then used by DAAP to compute an L-dictionary score for each segment in the dictionary set of segments and then the correlation between the L-dictionary scores and the judges’ scores was computed. The almost final dictionary was the L-dictionary that maximized this correlation. This maximal correlation, for n = 266, was about r = 0.75.

A few words, such as proper nouns (names) were too specific and were removed from the dictionary. However, there was low variation in the dictionary scores of the segments as computed by DAAP. It would be convenient to have them more spread out, and easier to read and understand. Using non-linear transformations, a series of adjustments was made to the dictionary weights to spread out the segment scores. These adjustments were:

1. The mean of the dictionary segment scores was adjusted to be at 0.
2. The positive weights were adjusted so that there was an item in the dictionary with weight + 1.
3. The negative weights were adjusted so that there was an item in the dictionary with weight − 1.
4. Most of the dictionary scores were close to 0. The positive weights were spread out with the weights closer to 0; more spread out than those closer to 1.
5. The negative weights were correspondingly spread out.

These transformations all preserve the order of the weights in the dictionary; that is, if the weight of word *a* was greater than the weight of word *b* before the adjustments, the weight of word *a* was still greater than the weight of word *b* after the adjustments.

We tested correlations to check whether these adjustments affected the validity. Before these adjustments to the weights, the correlation between the dictionary scores and the judges’ scores of the dictionary segments was r = 0.7552. After these adjustments, the correlation was n = 266, r = 0.7004. More to the point, the correlation between the adjusted dictionary scores and judges’ scores for the test segments was n = 30, r = 0.735, and the coverage was 0.812.

It is sometimes convenient to have dictionary weights and subsequent segment scores on a scale of − 1 to + 1, with 0 as the neutral value. It is also sometimes convenient to have these weights and scores on a scale of 0 to 1, with 0.5 as the neutral value. A simple linear transformation, y = (x + 1) / 2 takes the system with scores between -1 and + 1 to the system with scores between 0 and 1; its inverse, y = 2x − 1 does the reverse. I assume we can live with this dual scoring system just as the physicists have largely learned to live with the dual vision of light, as both a wave and a particle.

## The Concept of Power

The power of a word in a dictionary is the product of its weight and its frequency. The following tables show the ten most powerful positive and ten most powerful negative words in the WRAD, the WRRL, and the WRSL (Tables 1 and 2).

**Table 1:** Most powerful positive words in the WRAD, WRRL, and WRSL

WRAD				WRRL				WRSL			
Word	Wt	Freq	Power	Word	Wt	Freq	Power	Word	Wt	Freq	Power
and	1.0	0.051	5.067	I	0.094	0.082	0.768	I	0.220	0.019	1.85
the	1.0	0.037	3.707	that	0.177	0.030	0.511	mm	0.255	0.004	0.435
she	1.0	0.024	2.437	to	0.128	0.034	0.439	that	0.155	0.026	0.415
was	1.0	0.019	10.901	it	0.138	0.027	0.376	t^a^	0.195	0.004	0.404
in	1.0	0.013	1.483	you	0.139	0.024	0.333	know	0.300	0.010	0.314
a	0.625	0.022	1.377	is	0.323	0.009	0.312	don^a^	0.262	0.012	0.313
her	1.0	0.011	1.058	this	0.351	0.007	0.260	about	0.475	0.006	0.308
he	0.625	0.014	0.864	me	0.211	0.011	0.237	to	0.088	0.030	0.259
to	0.25	0.033	0.832	of	0.105	0.021	0.224	what	0.343	0.007	0.252
had	1.0	0.007	0.699	my	0.171	0.011	0.193	guess	0.715	0.003	0.231

^a^The single letters *t* and the item *don* refer to elements of the contraction don’t

**Table 2:** Most powerful negative words in the WRAD, WRRL, and WRSL

WRAD				WRRL				WRSL			
Word	Wt	Freq	Power	Word	Wt	Freq	Power	Word	Wt	Freq	Power
I	− 0.75	0.06	− 4.67	she	− 0.218	0.008	− 0.17	and	− 0.222	0.033	− 0.743
s^b^	− 1.0	0.026	− 2.57	we	− 0.28	0.005	− 0.138	the	− 0.293	0.021	− 0.616
it	− 0.875	0.026	− 2.28	he	− 0.205	0.005	− 0.103	he	− 0.633	0.007	− 0.457
that	− 0.875	0.023	− 2.02	go	− 0.267	0.003	− 0.079	so	− 0.377	0.011	− 0.380
t^b^	− 0.625	0.019	− 1.19	was	− 0.04	0.019	− 0.077	it	− 0.11	0.029	− 0.310
of	− 0.625	0.019	− 1.16	went	− 0.509	0.001	− 0.062	this	− 0.457	0.007	− 0.310
mm	− 0.625	0.014	− 0.89	like	− 0.635	0.001	− 0.06	at	− 0.487	0.005	− 0.244
is	− 1.0	0.009	− 0.89	had	− 0.088	0.006	− 0.051	we	− 0.443	0.005	− 0.235
you	− 0.625	0.012	− 0.727	said	− 0.182	0.003	− 0.05	out	− 0.632	0.004	− 0.223
what	− 1.0	0.007	− 0.726	they	− 0.09	0.005	− 0.042	is	− 0.254	0.008	− 0.191

^b^The single letters *s* and *t* refer to elements of contractions; the letter *s* represents the word *is*; the letter *t* represents the word not

The positive WRAD list and the negative WRRL list have 3 words (‘she,’ ‘he,’ ‘was’) in common. The positive WRRL list and the negative WRAD list have 5 words in common (‘I,’ ‘that,’ ‘is,’ ‘you,’ ‘it’); ‘I’ and ‘that’ are also on the list of positive WRSL words; ‘it’ is also on the negative WRSL list. The different dictionaries were made at different times in different places using different judges for different segments. We note that this phenomenon of several words being on both the positive WRRL list and the negative WRAD list does not occur in Italian; we are exploring the reasons for this.

## Implementation: History of DAAP

The first version of DAAP was written in 2002 in Visual Basic under the direction of Andres Roussos in Buenos Aires. I needed an experimental version and wrote a version in C +  + .

Following a suggestion of Sean Murphy’s, I wrote a version in Perl, using their regular expressions machine; then switched to Python. Both DAAP11 and TDAAP are written in Python.

The latest version of DAAP was written in MatLab in Bergamo, under the direction of Attà Negri.

## How DAAP Uses the Dictionaries: Smoothing and Foldovers

**Fig. 1:**
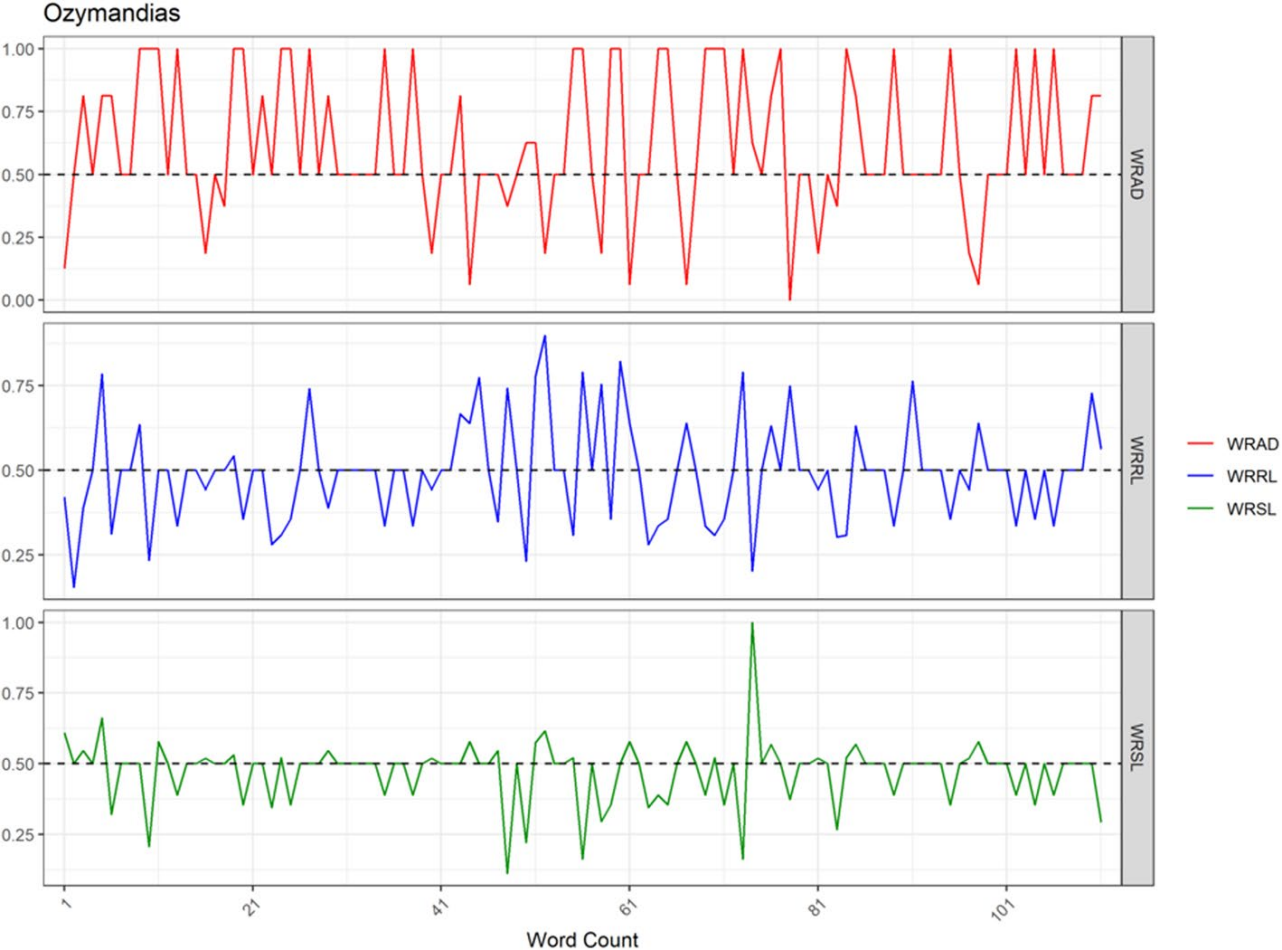
Unsmoothed language style measures for *Ozymandias*

Here are graphs of the words of the unsmoothed RP functions for the sonnet Ozymandias, by Shelley (Fig. 1).

I met a traveler from an antique land

Who said: Two vast and trunkless legs of stone

Stand in the [20] desert. Near them, on the sand,

Half sunk, a shattered visage lies, whose frown,

And wrinkled lip, and sneer of [40] cold command,

Tell that its sculptor well those passions read

Which yet survive, stamped on these lifeless things,

The hand [60] that mocked them and the heart that fed:

And on the pedestal these words appear:”

My name is Ozymandias, king [80] of kings:

Look on my works, ye Mighty, and despair!”

Nothing beside remains.

Round the decay of that colossal wreck, [100] boundless and bare

The lone and level sands stretch far away.

**Fig. 2:**
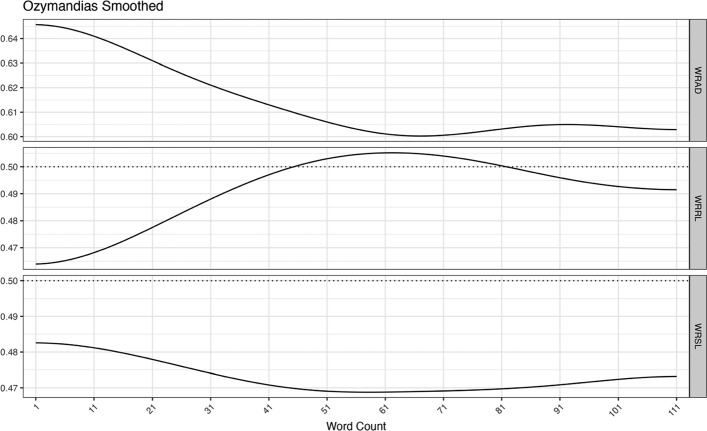
Smoothed language style measures for *Ozymandias*

As one can see in the above example, it is not easy to see patterns from these raw data. However, such patterns are easily discernible from the smooth data. Looking at the smoothed version of ‘Ozymandias’ as shown in Fig. 2, we can see a pattern emerge in the nature of the language that is used. The description of the statue is completed, and the emotional meanings are expressed.

CAUTION: Dangerous curves ahead. We have *weighted dictionaries* and *weighted averages*. The basic ingredient of the smoothing is a *moving weighted average* with an *exponential weighting function*. [Seat belts on.]

### What is a Weighted Average?

In understanding a weighted average, we will need to start with a formula for the average of a set of numbers. The mean or average, *M,* of a set of numbers, *x,* can be notated as x_J_, j = 1…, n, where j is the element’s position in the set and n represents the length of the set. From here, we set the formula as $M = \frac{{\sum\limits_{j} {x_{j} } }}{n}$., sum all the numbers in the set, and divide the sum by the length of the set.

In our case, where we think of the scores x_j_ as coming in a particular order,..., x_−m_,..., x_-2,_ x_−1_, x_0_, x_1_, x_2_..., the weighted average, with the weighting function, W^1^ is given the formula: $\frac{{\sum\limits_{j} {W_{j} } x_{j} }}{{\sum\limits_{j} {W_{j} } }}$, we multiply each number in the list with the corresponding weight, then take the average of those numbers.

**Fig. 3:**
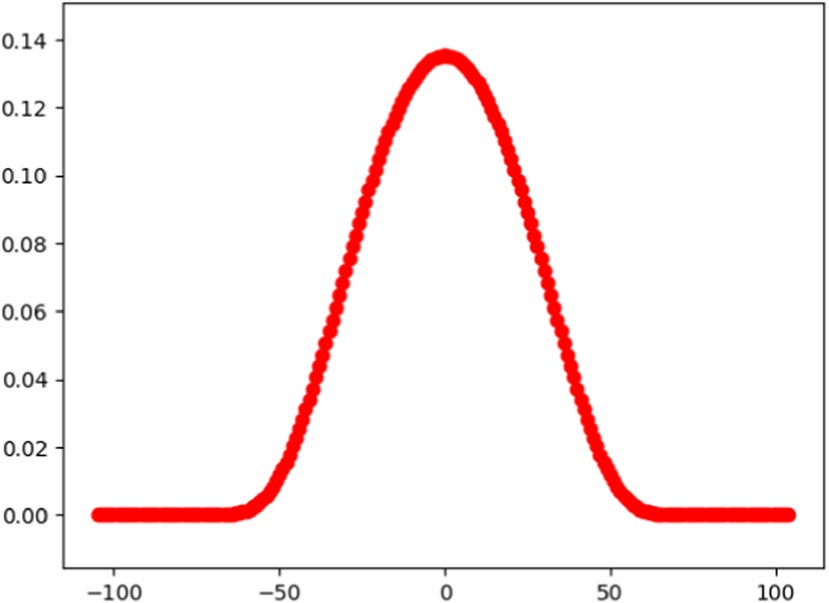
Weighting function

In order for this to be useful, we need the weighting function to have a particular shape. The weighting function, W, depends on two parameters: q and m. The parameters we use for DAAP are q = 2 and m = 100; the weighting function W is shown in Fig. 3.

Two points to notice:

1. W(j) is greatest at j = 0, then diminishes as the index j increases or decreases.
2. W is an even function; that is, W(− j) = W(j) for all j.

## The Moving Weighted Average

This moving weighted average can be readily applied for the middle of a turn, but how do we deal with the beginning and end of turns where there aren’t enough values to apply to the weighted average? The answer is a mathematical trick where we pretend that the dictionary values are folded over and reproduced ad infinitum in both directions. Then the moving weighted average makes sense at every word, including the first and last word. The smooth dictionary function is this moving weighted average, taken for every word of the turn.

That is, if the dictionary values for the words are, V_1_,..., V_n_, these imaginary values are given by, V_0_ = V_1_, V_−1_ = V_2_,..., V_n+1_ = V_n_, V_n+2_ = V_n−1_, etc.

Mathematical Fact: The mean of the smooth dictionary function is equal to the mean of the original dictionary function. This is true independent of the values of the parameters, q and m.

## The Mean Dictionary Value: The Effects of Word Order

The point of the above fact is that mean dictionary usage alone does not give us a lot of information about segments of text, including turns of speech or whole sessions. Mean dictionary usage is the main fact that most text analysis systems report. This includes the LIWC and other popular systems. A particular value of the weighting system developed for DAAP is that measures can be developed that allow for effects of word order, beyond the effects of the words alone.

Here are two segments, (turns of speech), which refer to the same event, use the exact same words, but in different order and to different effect. This difference in effect can be seen in the graphs and can be captured by other derived measures, as will be discussed.

**Fig. 4:**
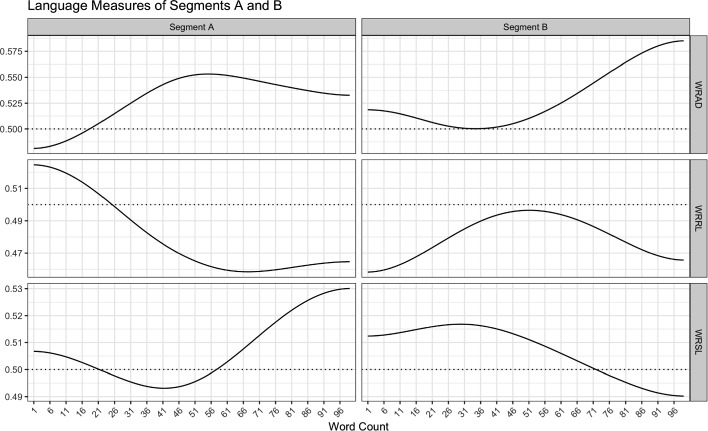
Effects of word order

Figure 4 shows the graphs for the two versions of the same event that differ only in word order.

*Segment A* mm, that just makes me think again of the feeling i find very hard to describe of, that was certainly stronger [20] in the few weeks right after i came out of the hospital, than it is now. but of when i was [40] nursing her and looking at her, and almost looking at her, looking at her, thinking, i’ve got to be [60] able to see her, and feeling i just couldn’t. and i suppose that was partly because i thought by [80] looking at her, i’d feel a warm feeling toward her, and i just really couldn’t.

*Segment B* right after i came out of the hospital, i just couldn’t. and i suppose that was partly be cause i [20] thought by looking at her, i’d feel a warm feeling toward her, and i just really couldn’t. mm, [40] that just makes me think again of (sniff) the feeling i find very hard to describe of, that was certainly stronger [60] in the few weeks than it is now. but of when i was nursing her and looking at her and [80] al most looking at her, looking at her, thinking, i’ve got to be able to see her, and feeling …

## Numerical Comparisons Based on Derived Measures

We have developed several new measures to be applied to whole segments of text, including turns of speech and entire transcripts. These depend on the DAAP smoothing function and are particularly useful for demonstrating the effects of word order.. The derived measures, Mean High Dictionary Value, High Dictionary Value Proportion, and the covariations all depend on the parameters M and q; for this reason, we keep those parameters fixed at M = 100, q = 2, for ordinary DAAP. (However, we use different values of these parameters for Time DAAP, as I’ll discuss later.)

Mean High WRAD is the mean amount by which the Smooth WRAD score is greater than the neutral value, where the mean is taken over all words for which the smooth WRAD is actually greater than 0.5. The High WRAD Proportion is the proportion of words for which this is true; that is, it is the proportion of words in the text for which the smooth WRAD curve is greater than the neutral value. The same explanations apply for the other weighted dictionaries WRRL and WRSL. I’ve discussed these measures in detail in a previous issue of this journal (Maskit, 2021).

We can compare the derived measures for the two texts shown above, covering the same event with the same words but with different word order, as shown in Table 3.

**Table 3:** Derived language style measures for segments A and B

Measure	Segment A	Segment B
Words	99	99
MWRAD	0.529	0.529
MHWRAD	− 0.538	0.029
HPWRAD	0.818	1.0
MWRRL	0.480	0.480
MHWRRL	0.016	0.000
HPWRRL	0.253	0.000
MWRSL	0.507	0.507
MHWRSL	0.013	0.012
HPWRSL	0.636	0.717
WRAD/WRRL	− 0.956	− 0.358
WRAD/WRSL	0.062	− 0.986
WRRL/WRSL	− 0.350	0.208

While the basic dictionary measures do not differ for these texts, the variation in word order produces notable differences for the derived measures, and most strongly for the covariations.

## Some New Directions Using DAAP

### Producing New Dictionaries

There are many possible new avenues to explore using the opportunities provided by DAAP. For example, here is another passage of text that receives a high WRAD score: well, it was like i got up this morning and i was going to meet my manager downtown, you know, early this morning, you know. it was like, i looked at the clock and it was six forty-five and i thought. i usually get up about five to seven or seven and i thought to myself, you know, if i wait until five to seven or seven o’clock i’m going to get up and i going to say to myself that i don’t have time to take a shower and get ready before i come here to talk to you and so it will just make me even later getting downtown. so, i thought to myself, i’ll get up right now, take my shower and shave and get ready and go straight down to work from when i get out of here, and it didn’t take a lot of effort to do that.

Is it my idiosyncratic response to this segment that it is not Symbolizing, but something else, primarily boring? It would be good if we had a Symbolizing dictionary that distinguished Symbolizing from blathering.

Each of the current affect dictionaries is a hodgepodge of words related to positive, negative, and neutral affect but without any distinction of the different possible relationships of these words to what the speaker or writer was actually feeling in the moment of speaking. Could one or more weighted dictionaries be developed that more accurately pick up a speaker’s expression of their current feelings, such as the extent of emotional involvement in an experience, as well as its valence?

## The Time DAAP (TDAAP)

We now move on to a major new aspect of our approach to language analysis, one that brings us even closer to representing the relationship between emotion and its verbal expression. Among her other requests, Wilma Bucci also challenged me to produce a version of DAAP based on a timeline rather than a sequence of words. This would be a way to have our measures represent not only the words but the rhythms of spoken language, and the interaction patterns between speakers. In addition, the production of time-based measures would allow the application of an acoustic analysis of intonation patterns using software such as PRAAT. Such a program would enable a more complete application of the basic principle of Multiple Code Theory—the relation of bodily experience to the expression of emotional experience in language. The relationship between paralinguistic features of speech and language-style and content; the nature of non-symbolic communication between patient and therapist; and the dynamics of mutual influence in conversation are just a few of the possible areas of study that a version of DAAP based on time can open to investigation within the context of the Referential Process.

We now have such a program, the Time DAAP (TDAAP), which I’ll outline briefly here^2^:

1. Utilizing Google Cloud’s Speech-To-Text API, a Python program was created to both apply Google’s speech-to-text and output the necessary data.

2. The Python program extracts the word, time marker, and speaker for every word and outputs the data into a text file.

3. The text file is then run through another program we created called preTDAAP, which converts the data into a CSV spreadsheet.

4. The spreadsheet is then proof-listened by a human, correcting, among others, words marked as of zero duration, and so forming word blocks.

5. TDAAP reads the word blocks in the corrected CSV file and separates the word blocks into utterances according to the length of pauses.

6. An utterance is defined by a gap or pause of at least one half second between the end of one word block and the beginning of the next.

7. TDAAP produces the GAP (Pause) file. This file shows number and mean length of pauses between each pair of speakers and within the turns of each speaker.

8. TDAAP produces the marked text (MTT File), which reproduces the text and inserts markers every 5 seconds and every 20 words.

9. A mean dictionary score is produced for each dictionary and each word block.

10. The dictionary values are smoothed separately for each utterance.

## How Long is a Pause?

Spoken language is marked by a myriad of different kinds of silences. Some of these silences are intentional pauses conveying demonstrable and explicit meanings, while others communicate implicit meaning. A long pause is sometimes an invitation for the other person to speak; it can signify a change in a speaker’s direction. But how do you draw the line between short and long pauses? We have chosen one half-second as the dividing line, but this is an arbitrary choice. (See Peterson, this issue, for more detail on this aspect of the program.) Some pauses, too short to be registered consciously, may convey information at a level of processing that occurs outside of awareness. The length of a pause can be adjusted to the tenth of a second; future research using the TimeDAAP may identify pauses of different lengths as operative in various inter-, and intrapersonal psychological processes.

## From Dictionary Values to Time; How Do We Assign Dictionary Values to Time?

Each block of words, as defined by the procedures described above, contains some number of words and has a duration of some number of milliseconds. Each word in the block has a dictionary score for each dictionary in use. The dictionary score for the block is the mean of the dictionary scores for the individual words in the block. The WRDUTT File records this basic data for each utterance. Then, the SMTUTT File records the data for the smooth dictionary values.

### Volubility

TDAAP produces a new concept of volubility measured as Words per Second (WPS). This measure is directly computed (number of words times 10, divided by the duration in number of milliseconds) for each word block, and then smoothed across each utterance.

### Pause Data

The pause file shows the number and mean length of pauses (of length at least one-half second) within the turns of each speaker, and between speakers.

## How do we Smooth Over Time?

Each utterance contains some number of blocks, each of some measurable duration. There are also some number of small pauses (less than 1/2 s) between blocks. For the time within each block, each half second gets the dictionary score of the block. For the time between blocks, the dictionary scores at the half-second intervals between blocks are filled in linearly. These dictionary scores are then smoothed across the entire utterance.

In addition to the basic demographic data, the WRDUTT and SMTUTT files contain the following data for each utterance:

1. The time points; i.e., the x-axis data points.

2. Mean WPS (Words Per Second).

3. Mean Dictionary score for each unweighted dictionary.

4. Mean dictionary score for each weighted dictionary.

5. Mean High Dictionary Value and High Dictionary Proportion. These are basically the same as the usual measures, except that they are computed by time; that is, instead of there being a computation at every word, there is a computation at every half second.

6. The covariations are similarly computed with comparative data every half second.

7. The absolute covariation, to be discussed below, is a new measure using time and the weighted dictionaries.

## Absolute Covariations

Originally, the covariation was designed to be, computationally, the same as the correlation coefficient, but given a different name, with different statistical parameters, as the smooth dictionary data at nearby words are not statistically independent. The covariation is the measure of the extent to which the two variables rise and fall simultaneously. This requires the designation of a “zero” point for each measure, and a priori there is no such point, so correlation and covariation use the mean of the sample for each variable as the “zero” point.

However, the weighted dictionaries all have the neutral value, or zero point built in, so the absolute covariation, rather than asking to what extent the two variables are simultaneously above their own mean, asks to what extent the two variables are simultaneously above the built-in neutral value.

## Aggregate Data

The final steps in the current version of TDAAP are first, to aggregate the half second data (WPS, Unweighted Dictionaries, Weighted Dictionaries) by speaker for each file (usually a session), providing the data for graphing these data for each file or session; and second, to provide summary data (Mean WPS; Mean Dictionary Values; Mean High Dictionary Values; High Dictionary Proportions; Covariations between each pair of dictionary variables, and Absolute Covariations between each pair of weighted dictionaries) for each speaker and for each file or session.

## Work in Progress for TDAAP

1. The TDAAP measures, including WPS and Absolute Covariations, have not yet been tested. We have one data set ready to be run and we have recordings for two more data sets.

2. The second phase of TDAAP (matching linguistic data with voice qualities such as loudness and pitch) needs experimentation.

3. TDAAP needs to be adapted for Italian, Spanish, and other languages.

## Data Availability

The data that support the findings of this study are available from the corresponding author, WB, upon reasonable request.

## Footnotes

1 The weighted function for a given value *m*, $W\left(j\right)=\frac{{e}^{-2{m}^{2}}\frac{{m}^{2}+{j}^{2}}{{({m}^{2}-{j}^{2})}^{2}}}{K}$, where $K= {\sum }_{x=1-m}^{m-1}{e}^{-2{m}^{2}}\frac{{m}^{2}+{x}^{2}}{{({m}^{2}-{x}^{2})}^{2}}$ for integers lying between 1-*m* and *m-1* inclusive and W(j) = 0 for values of *j* beyond the interval.

2 The editors note that this presentation at the Referential Process Workshop by Bernie Maskit is the only formal presentation of the TDAAP procedures that has been shared. Some aspects of the method and an application are included in the paper by Henry Peterson in this issue.

## References

[CR1] Bucci, W., Kabasakalian, McKay, R., the RA Research Group (1992). Instructions for scoring Referential Activity (RA) in transcripts of spoken narrative texts. In Ulm, Germany; Ulmer Textbank

[CR2] Bucci, W., McKay, R.K., (2014): Manual for scoring RA scales. In (Original publication, 1992) figshare. 10.6084/m9.figshare.962956

[CR3] Bucci, W. (1984). Linking words and things: Basic processes and individual variation. *Cognition,**17*, 137–153. 6541111 10.1016/0010-0277(84)90016-7

[CR4] Bucci, W. (1997). *Psychoanalysis and cognitive science: A multiple code theory*. Guilford Press.

[CR5] Bucci, W. (2021). *Emotional communication and therapeutic change: Understanding psychotherapy through multiple code theory*. Routledge.

[CR6] Bucci, W., & Freedman, N. (1978). Language and hand: The dimension of referential competence. *Journal of Personality,**46*, 594–622.

[CR100] Bucci, W. & Maskit, B. (2006) A weighted dictionary for Referential Activity. In J.G. Shanahan , Y. Questionnaire, & J. Wiebe (Eds.) Computing Attitude and Affect in Text; Dordrecht, The Netherlands: Springer; pp. 49-60

[CR7] Mariani, R., Maskit, B., Bucci, W., & De Coro, A. (2013). Linguistic measures of the referential process in psychodynamic treatment: The English and Italian versions. *Psychotherapy Research: Journal of the Society for Psychotherapy Research,**23*(4), 430–447. 10.1080/10503307.2013.794399 23656534 10.1080/10503307.2013.794399

[CR8] Maskit, B. (2021). Overview of computer measures of the referential process. *Journal of Psycholinguistic Research,**50*(1), 29–49. 10.1007/s10936-021-09761-8 33464426 10.1007/s10936-021-09761-8

[CR9] Mergenthaler, E., & Bucci, W. (1999). Linking verbal and non-verbal representations: Computer analysis of referential activity. *British Journal of Medical Psychology. United Kingdom: British Psychological Society.*10.1348/000711299160040 10.1348/000711299160040 10524719

[CR10] Peterson, H. (2025). Changes in speech rhythm and language-style between in-person and remote treatment. In Journal of psycholinguistic research, XX(X), XX-XX. 10.1007/s10936-025-10157-1 40402177

[CR11] Strunk, W., Jr., & White, E. B. (1972). *The elements of style*. The Macmillan Co.

[CR12] Tocatly, K. (2025). Characterizing the language of therapist interventions in moments of high patient Arousal. In Journal of psycholinguistic research, XX(X), XX-XX. 10.1007/s10936-025-10164-2 40789794

[CR13] Zhou, Y., Maskit, B., Bucci, W., Fishman, A., & Murphy, S. (2021). Development of WRRL: A new computer measure of the Reflecting/Reorganizing function. *Journal of Psycholinguistic Research,**50*(1), 51–64. 33511546 10.1007/s10936-021-09762-7

